# ZnO Nanoparticles Induce Dyslipidemia and Atherosclerotic Lesions Leading to Changes in Vascular Contractility and Cannabinoid Receptors Expression as Well as Increased Blood Pressure

**DOI:** 10.3390/nano11092319

**Published:** 2021-09-07

**Authors:** Adriana Ceballos-Gutiérrez, Alejandrina Rodríguez-Hernández, María del Rosario Álvarez-Valadez, Saraí Limón-Miranda, Felipa Andrade, Alejandro Figueroa-Gutiérrez, Irene Díaz-Reval, Alejandro Apolinar-Iribe, Luis Castro-Sánchez, Javier Alamilla, Enrique Sánchez-Pastor, Adolfo Virgen-Ortiz

**Affiliations:** 1 Facultad de Medicina, Universidad de Colima, Colima 28040, Mexico; aceballos12@ucol.mx (A.C.-G.); arodrig@ucol.mx (A.R.-H.); afigueroa3@ucol.mx (A.F.-G.); 2 Centro Universitario de Investigaciones Biomédicas, Universidad de Colima, Colima 28045, Mexico; Malvarez52@ucol.mx (M.d.R.Á.-V.); idiazre@ucol.mx (I.D.-R.); 3 Departamento de Ciencias Químico Biológicas y Agropecuarias, Unidad Regional Sur, Universidad de Sonora, Navojoa 85880, Mexico; Sarai.limon@unison.mx; 4 Institito Tecnológico de Colima, Colima 28076, Mexico; Felipa.andrade@colima.tecnm.mx; 5 Departamento de Física, Unidad Centro, Universidad de Sonora, Hermosillo 83000, Mexico; apolinar@ciencias.uson.mx; 6 Centro Universitario de Investigaciones Biomédicas, CONACYT-Universidad de Colima, Universidad de Colima, Colima 28045, Mexico; Luis_castro@ucol.mx (L.C.-S.); javier_alamilla@ucol.mx (J.A.)

**Keywords:** ZnO nanoparticles, dyslipidemia, cardiovascular, atherosclerosis, vascular contractility, blood pressure, cannabinoid receptor, CB_1_ receptor, CB_2_ receptor

## Abstract

ZnO nanoparticles (ZnONPs) have been shown to have therapeutic potential in some diseases such as diabetes and cancer. However, concentration-dependent adverse effects have also been reported. Studies which evaluate the effects of ZnONPs on the cardiovascular system are scarce. This study aimed to evaluate the cardiovascular effects of a low dose of ZnONPs administered chronically in healthy rats. Changes in dyslipidemia biomarkers, blood pressure, aortic wall structure, vascular contractility, and expression of cannabinoid receptors in the aorta wall were evaluated. Healthy rats were divided into two groups: control or treated (one, two, and three months). The treated rats received an oral dose of 10 mg/kg/day. The results showed that treatment with ZnONPs induced dyslipidemia from the first month, increasing atherosclerosis risk, which was confirmed by presence of atherosclerotic alterations revealed by aorta histological analysis. In in vitro assays, ZnONPs modified the aorta contractile activity in response to the activation of cannabinoid receptors (CB_1_ and CB_2_). The expression of CB_1_ and CB_2_ was modified as well. Moreover, ZnONPs elicited an increase in blood pressure. In conclusion, long-time oral administration of ZnONPs induce dyslipidemia and atherosclerosis eliciting alterations in aorta contractility, CB_1_ and CB_2_ receptors expression, and an increase in blood pressure in healthy rats.

## 1. Introduction

Zinc oxide nanoparticles (ZnONPs) are being studied widely due to their multiple applications. Previous works on biological systems show that ZnONPs have antibacterial, antifungal, antiviral, anticancer, and antidiabetic activity. In the field of engineering, it has been reported that ZnONPs are useful in solar cells, gas sensors, chemical sensors, biosensors, and photodetectors [1]. The potential of therapeutic application of these nanoparticles is promising for the treatment of chronic diseases such as diabetes mellitus [2,3,4,5,6,7]. Thus, more research is needed to evaluate in detail their biological and toxicological effects in different tissues after long periods of administration. The effect of ZnONPs depends on their physicochemical properties, exposure time, dose or concentration, pH, and biocompatibility. ZnONPs have been reported to release zinc ions capable of inducing oxidative stress and cell damage, generating a cytotoxic and genotoxic effect. Although there are studies that have evaluated the effects of ZnONPs on some organs or systems, the current knowledge is limited. Toxicity studies mainly at high doses of ZnONPs have shown accumulation of zinc in the liver, kidney, lung, heart, and spleen, inducing injury and in some cases death [8]. Other studies also showed this type of effect was induced by ZnONPs, but dependent on the route of administration [9]. Hematological alterations, stomach, pancreas, and retina injuries have also been reported [10] and, more recently, alterations in the nervous system [11,12].

In this context, recent studies have evaluated the effects of ZnONPs on the cardiovascular system reporting interesting observations [13,14]. First, in an in vitro test with human coronary artery endothelial cells, they observed inflammation and an increase in cell adhesion molecules expression in a concentration-dependent manner. They later observed in an in vivo trial that ZnONPs induce dyslipidemia, systemic inflammation, and an increase in the thickness of the aortic wall, suggesting that ZnONPs can produce atherosclerotic alterations at very low doses administered intratracheally [13]. In contrast, another study carried out in healthy rats showed that low doses of ZnONPs administered intragastrically did not affect the metabolic biomarkers that evaluate atherogenic risk. However, at doses of 10 mg/kg, systemic inflammation was observed [14].

The lack of knowledge of the ZnONPs’ effects on the cardiovascular system, as well as the discrepancies in the risk of generating atherosclerosis, made us propose a study to evaluate the effects of a low dose of ZnONPs administered chronically on blood pressure, structure of the aortic wall, dyslipidemia biomarkers, and vascular contractility. On the other hand, pathophysiological studies carried out to better understand the mechanisms involved in atherosclerosis suggest an important role of the endocannabinoid system in the progression of this disease [15,16,17,18]. Thus, it was also in our interest to evaluate in this study whether the atherosclerotic alterations induced by ZnONPs are associated with changes in the expression of CB_1_ and CB_2_ cannabinoid receptors in the aorta wall.

## 2. Materials and Methods

### 2.1. Animals

Male rats of the Wistar strain with an average weight of 310 ± 10 g were used for the study. All the animals were kept in a room with controlled temperature conditions (24 °C) and 12:12 h of light-dark cycles. During all experimental protocols they had free access to water and food. All the procedures used were approved by the bioethics committee of the University Center for Biomedical Research of the University of Colima (Project code 2020-1; approval date: 12 February 2020).

### 2.2. Experimental Design

Sixty rats were randomly divided into two experimental groups: control (*n* = 30) and treated with ZnONPs (10 mg/kg, *n* = 30), then each group was subdivided into three groups (*n* = 10 per group) corresponding to one, two, and three months of treatment.

### 2.3. ZnONPs Preparation

ZnONPs dispersion was purchased from Sigma-Aldrich (catalog number 721077, St. Louis, MO, USA). For the treatment, a fresh dispersion was prepared at a concentration of 5 mg/mL diluted in 0.9% sodium chloride solution. In order to have a homogeneous dispersion, the mixture was sonicated in an ultrasonic bath with pulse cycles of 30 s and 30 s of rest, and a pulse amplitude of 50%. Freshly dispersed the ZnONPs were administered intragastrically with a daily dose of 10 mg/kg for one, two, and three months, corresponding to each experimental group. The ZnONPs used in this study have a spherical shape, with an average size of 17 nm. These data were previously reported by our research group [19].

### 2.4. Measurement of the Blood Pressure in Rats

Before measuring the blood pressure, for a week the rats were adapted to the restrain tube and acclimatized to the experimentation room. On the day of the experiment, the measurement was carried out as follows: the rat was place into the restraining tube and holders inside tube were adjusted to prevent movement of the animal. Subsequently, a volume pressure recording sensor coupled occlusion cuff was placed on the rat tail base, and measurements were made using a non-invasive Mouse and Rat Blood Pressure System (IITC Life Science Inc, Woodland Hills, CA, USA). Five recordings were made with a 1-min rest between each recording, and the average of the blood pressure was calculated for each rat. This equipment used to measure blood pressure has a high reliability photoelectric sensor to detect pulsation at a room temperature of 32 °C or less, avoiding stress to the rat and alterations in blood flow due to excessive heat. The rat tail temperature was recorded during the blood pressure measurement (30 ± 1.5 °C).

### 2.5. Biochemical Determinations

Blood samples were obtained by an intracardiac puncture at 30, 60, and 90 days from each experimental group, then the serum was separated by centrifugation at 3000 rpm for five min. The serum was aliquoted and stored at −80 °C until later analysis. Following the protocols recommended by the manufacturer (SPINREACT, Girona, Spain), total cholesterol (TC), HDL-cholesterol (HDL-C), LDL-cholesterol (LDL-C), triglycerides (TG), as well as gamma-glutamyl transferase (GGT), alkaline phosphatase (ALP), aspartate aminotransferase (AST), alanine aminotransferase (ALT), and lactate dehydrogenase (LDH) enzymes were determined in the samples.

The atherogenic index was calculated from the equation: Log10 (TG/HDL-C).

### 2.6. Histological Analysis

In each experimental group, the descending aorta was removed, placed in a Sylgard-coated Petri dish filled with oxygenated Krebs-Henseleit (SKH) solution, to remove surrounding adipose and connective tissue. A fragment of the aorta was fixed in 10% buffered formalin, embedded in paraffin, cut (5 μm sections), and stained with the hematoxylin-eosin technique for histological analysis. Another fragment of the aorta was used for the study of vascular contractility, and the immunohistochemical analysis.

### 2.7. CB_1_ and CB_2_ Receptors Expression in Aorta Rings by Immunofluorescence

CB_1_ and CB_2_ receptor expression in the aorta wall was studied immunohistochemically followed by confocal microscopy. Cross sections (2 mm) of the aorta were fixed in 4% paraformaldehyde, embedded in paraffin, cut, place on a slide, deparaffinized, and rehydrated. Next, aorta sections were blocked (Animal-free blocker and diluent, Vector laboratories Inc., Burlingame, CA, USA) and incubated with rabbit polyclonal Anti-CB_1_ (1:100, Alomone, Labs, Jerusalem, Israel) or rabbit polyclonal Anti-CB_2_ (1:100, Alomone, Labs, Jerusalem, Israel) primary antibody and co-labeled with mouse monoclonal Anti-alpha smooth muscle Actin antibody (1:200, Abcam, Cambrige, UK) overnight. The next day, primary antibodies were removed, and the samples were incubated with secondary antibodies for 2 h (FITC anti-rabbit, 1:100, Abcam (Cambrige, UK); and Alexa Fluor-568 anti-mouse, 1:50, Thermo Fisher Scientific, Waltham, MA, USA). Nuclei were counterstained with DAPI. Then a coverslip was placed using an antifade reagent (Prolong Diamond Antifade, Invitrogen, Waltham, MA, USA). Finally, images were acquired using a confocal microscope (Mod LSM 700, Zeiss), 3-D blind deconvolved (AutoQuant X3, Media Cybernetics), and subsequently analyzed with ZEN2009 (Zeiss), and Image J software (National Institutes of Health, Bethesda, ML, USA).

### 2.8. Vascular Contractility

It is well documented that the cannabinoid receptors participate in the regulation of blood pressure at the vascular level. Therefore, to know if treatment with ZnONPs modifies the contractility at this level, the effects of selective agonists for both receptors, CB_1_ and CB_2_, on aorta contractility were evaluated in vitro. For these experiments, the following protocol was carried out: aortic rings approximately 2 mm wide were mounted in an isolated organ bath filled with Krebs-Henseleit solution (KHS) bubbled with carbogen (95% O_2_, 5% CO_2_). The ring was then fixed between two stainless steel wires, one of them coupled to an isometric force transducer (Radnoti) whose signal was amplified (CyberAmp 380, Axon instruments inc., Foster City, CA, USA) and digitized by an analog-digital interface (Digidata 1200, Axon instruments inc., Foster City, CA, USA). The initial tension of the ring was adjusted to 2 g and stabilized for 60 min. Then a contracture was generated with phenylephrine (1 μM) reaching its maximum response after approximately 15 min. At this point, the effect of the agonists ACPA 30 μM (selective for CB_1_ receptor) or HU308 10 μM (selective for CB_2_ receptor) on tension was evaluated. The tension records were acquired and analyzed with Axoscope and Clampfit software (Version 9, Axon Instruments Inc., Foster City, CA, USA), respectively.

The bath temperature was kept constant at 37 °C for the duration of the experiment. The composition of the KH solution was the following (in mM): 118 NaCl, 5 KCl, 1.2 MgSO_4_, 1.2 KH_2_PO_4_, 25 NaHCO_3_, 2 CaCl_2_, added with 2 g D-Glucose, pH 7.4.

### 2.9. Statistical Analysis

Data were expressed as means ± standard error. The experimental groups were analyzed using a one-way analysis of variance and the comparison between pairs of groups was performed with the Bonferroni test. The differences were considered significant with *p* < 0.05. All the processing and statistical analyses of the data were carried out with GraphPad Prism 8 software.

## 3. Results

### 3.1. Effects of the ZnONPs on Lipid Profile and Atherogenic Risk

The treatment with ZnONPs did not alter total cholesterol and serum HDL-C in healthy rats in the first 2 months. However, three months later, the total cholesterol and HDL-C increased significantly to 29% and 81% respectively compared to the control group (Table 1). ZnONPs decreased LDL-C concentration, an effect that was significant at the first (49%) and third month (26%) of the treatment compared to the control group. With respect to triglycerides concentration, the treatment significantly increased its levels after one (50%) and two months (34%) compared to the control. Finally, the atherogenic risk with the treatment of ZnONPs increased significantly only in the first month (240%) compared to the control group (Table 1).

### 3.2. Effect of the ZnONPs Treatment on the Structure of the Aorta Wall

**Figure 1:**
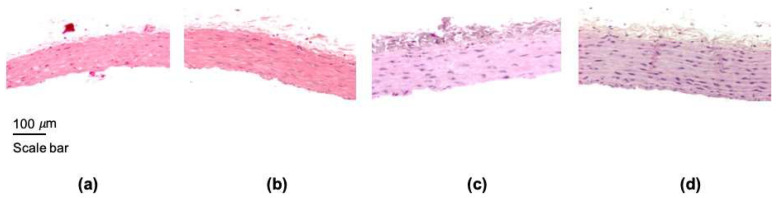
Representative images of aorta sections stained with hematoxylin-eosin. (**a**) control or healthy rat, (**b**) ZnONPs-One month treated rat, (**c**) ZnONPs-Two months treated rat, and (**d**) ZnONPs-Three months treated rat. All images were acquired with 10× magnification.

**Figure 2:**
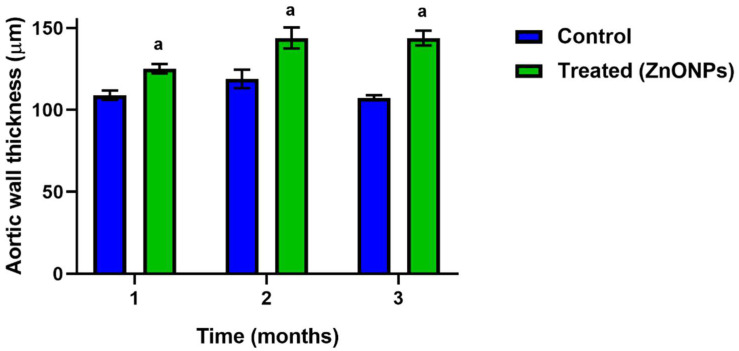
Effect of ZnONPs on the aortic wall thickness. Blue bars correspond to the control non-treated group; green bars correspond to the ZnONPs treated-groups at different times. a, there is a significant difference compared to the control group with *p* < 0.05.

The first parameter evaluated was the thickness of the aorta wall. The thickness significantly increased since the first month of the treatment and was maintained for three months as shown in Figure 1 and Figure 2.

**Figure 3:**
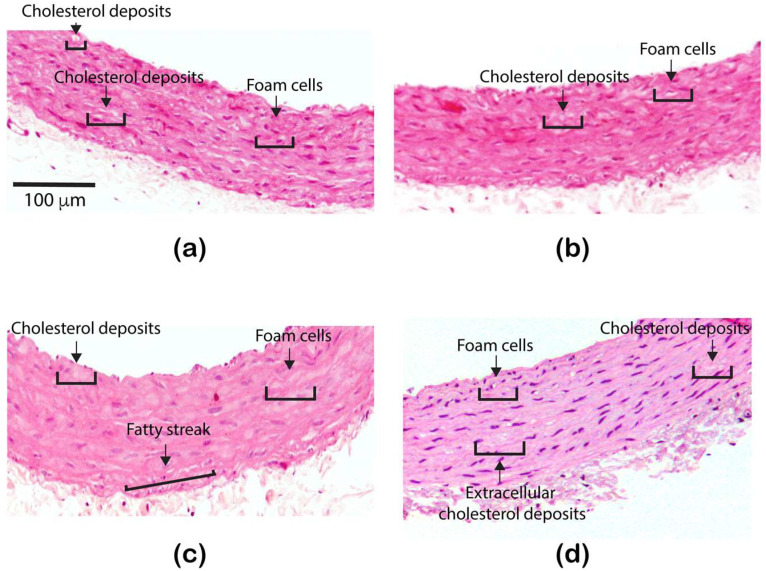
Illustrative images showing atherosclerotic lesions after of the treatment with ZnONPs: (**a**) control or healthy rat, (**b**) ZnONPs-1 month treated rat, (**c**) ZnONPs-2 months treated rat, and (**d**) ZnONPs-3 months treated rat. All images were acquired with 10× magnification.

The histological analysis in the slides stained with hematoxylin-eosin did not reveal differences in the first month of the treatment, foam cells and normal cholesterol drops were observed in both groups, control and treated (Figure 3a,b). Furthermore, in the second month, a greater accumulation of intracellular lipids was distinguished in the rats treated with ZnONPs, and in the third month, extracellular lipid deposits and fatty streaks were detected in the treated group (Figure 3c,d). The structural characteristics observed in the rat aorta after two or three months of the treatment with ZnONPs are typical in the atherosclerotic process.

### 3.3. Alterations in ZnONPs-Induced Vascular Contractility

**Figure 4:**
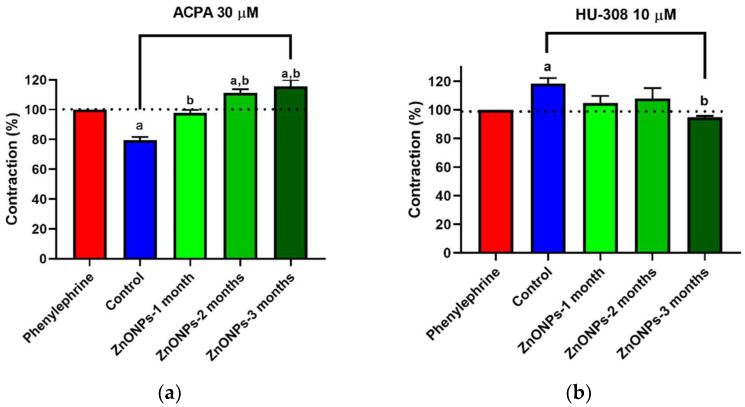
Effect of ZnONPs on aorta contractility. (**a**) Effect of ACPA (CB_1_ receptor agonist) on contraction in aortic rings in different experimental groups. (**b**) Effect of HU308 (CB_2_ receptor agonist) on contraction in aortic rings in different experimental groups. Blue bars correspond to the control non-treated group, green bars correspond to the ZnONPs treated-groups at different times; (**a**) there is a significant difference compared to the phenylephrine group with *p* < 0.05; (**b**) there is a significant difference compared to the control group with *p* < 0.05.

ACPA (CB_1_ receptor agonist) induced 20% vasorelaxation in the aorta of untreated rats (Figure 4a). Interestingly, ACPA in the aorta of rats that received ZnONPs for one month had no effect on the vascular tension. The treatment attenuated the vasorelaxation observed in control rats. At a treatment longer time with ZnONPs, ACPA induced vasoconstriction, 11% at two months and 16% at the end of the third month (Figure 4a).

When HU308 (CB_2_ receptor agonist) was evaluated on aorta contractile activity, a vasoconstrictor effect (19%) was observed in untreated rats. In ZnONPs-treated rats, the vasoconstriction effect HU308 was lower than that observed in untreated rats. The vasoconstriction was not statistically significant in the first two months. After the third month, the vasoconstriction was completely abolished, and 5% vasorelaxation was even observed (Figure 4b).

### 3.4. CB_1_ and CB_2_ Receptors Expression in Aorta Rings of ZnONPs-Treated Rats

**Figure 5:**
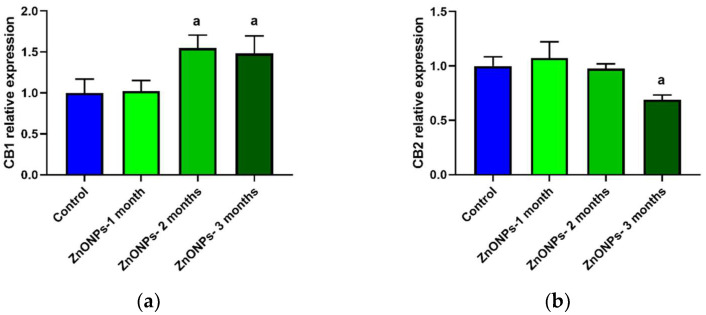
Effect of ZnONPs on the CB_1_ and CB_2_ receptors expression in the aorta wall. (**a**) CB_1_ expression, (**b**) CB_2_ expression. Blue bars correspond to the control non-treated group, green bars correspond to the ZnONPs treated-groups at different times; (**a**) there is a significant difference compared to the control group with *p* < 0.05.

To determine the expression of cannabinoid receptors in the aorta, the mean fluorescence intensity was quantified, and results were normalized concerning the control group that did not receive ZnONPs treatment. The first month of treatment with ZnONPs did not alter the expression of the CB_1_ receptor in the aorta wall (Figure 5a).

**Figure 6:**
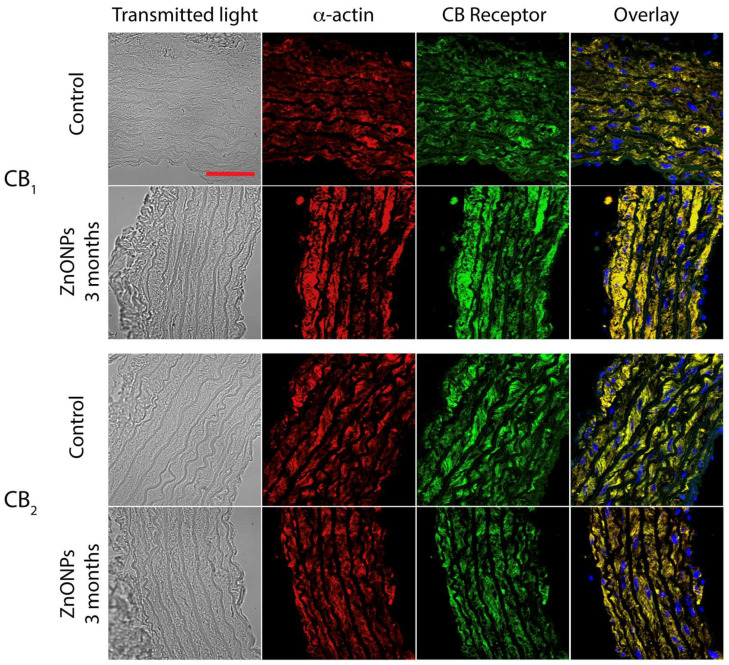
Representative images showed ZnONPs effect on CB_1_ and CB_2_ receptors expression in aorta wall. Transmitted light images (grey). Scale bar corresponds to 50 μm. Smooth muscle α-actin was detected with a specific antibody labeled with Alexa Fluor 568 (red). CB_1_ and CB_2_ located on the aorta ring were detected by specific antibodies and labeled with FITC (green). Image overlap indicates a high degree of colocalization of CB_1_ or CB_2,_ and smooth muscle α-actin (yellow). Nuclei were counterstained with DAPI dye (blue).

In contrast, after two and three months, the expression significantly increased, approximately 50% in comparison to the control group (Figure 5a). In the case of the CB_2_ receptor, ZnONPs did not modify the expression during the first two months of treatment, while after three months, the expression decreased significantly by 31% compared to the control group (Figure 5b) (See illustrative images in Figure 6).

### 3.5. ZnONPs on Blood Pressure

Systolic blood pressure (SBP) is a parameter used for the diagnosis of arterial hypertension. In this study, the oral administration of ZnONPs in healthy rats significantly increased SBP (18%) after three months in compared to the non-treated control group as shown in Table 2.

## 4. Discussion

In the present study, the effects of the long-term oral administration of ZnONPs (10 mg/kg) on lipid profile, blood pressure, vascular structure, and contractility, as well as on the expression of CB_1_ and CB_2_ cannabinoid receptors in healthy rats, were evaluated.

The results of the present research showed that ZnONPs induce changes in the serum lipid profile. They mainly increase triglycerides during the first two months of the treatment and total cholesterol and HDL-C after three months (Table 1). In addition, a high atherogenic risk was observed after one month of treatment. In the literature [13,20,21], it has been reported that ZnONPs induce lipid alterations depending on the dose or concentration, the administration route, and the exposure time. Intratracheal administration of ZnONPs (1.25–5 mg/kg) in rats for 12 weeks induced an increase in TC and LDL-C and a decrease in HDL-C, dependent on the concentration [13]. In another study also carried out in rats, after 2 weeks of administration (25–50 mg/kg) TG increased, and HDL-L and LDL-C decreased in a dose-dependent manner [20]. In healthy mice that received ZnONPs orally (333 mg/kg, 5 days), TG increased and both HDL-C and LDL-C decreased [21]. In contrast to the dyslipidemia observed in healthy subjects, recent studies reported that ZnONPs (5–10 mg/kg) decreased serum TC and TG in rats with hepatocellular carcinoma, improving their lipid metabolism [22], while in diabetic rats, ZnONPs at 3 mg/kg decreased TC, increased HDL-C and reduced the risk of atherosclerosis [14]. In general, dyslipidemia observed in healthy subjects increases atherogenic and cardiovascular risk. Increased atherogenic index the first month of the treatment in our experiments agree with other authors [13] and contrasts with another study that did not observe changes in the atherosclerosis development risk [14]. It is also important to say that although some genetic and molecular studies report homologies between cholesterol-transport lipoproteins in mice and humans [23,24], other studies have reported differences between rodents and humans in the lipid metabolism. While in humans LDL-C is the main responsible for the transport of cholesterol, in mice and rats it has been suggested that it is HDL-C [25,26]. These differences could help to improve the understanding of what was observed in our study where both total cholesterol and HDL-C increase in the second month and these differences become significant in the third month, which is consistent with the atherosclerotic alterations observed in the histological analysis. However, more studies are needed to understand the metabolic and functional implications of these reported differences between rodents and humans in the lipid metabolism.

We were also interested to know if this dyslipidemia induced by ZnONPs also elicited changes in the vascular structure. Our results showed that aorta wall thickness increased (Figure 1 and Figure 2), an observation that agrees with a previous study [13]. In addition, the results of the histological analysis revealed the formation of fatty streaks in the aorta wall (Figure 3), lesions that are very common of the atherosclerosis process. Our results support previous observations made in in vitro assays which suggest that ZnONPs can induce atherosclerosis and increase cardiovascular risk through an inflammatory mechanism [13,27,28], mediated by the formation of reactive oxygen species, ROS [29,30,31], and zinc ions playing a key role [32]. However, investigation to study the effects of ZnONPs at a dose of 10 mg/kg on oxidative stress and vascular inflammation in rats is needed.

This study is the first to analyze the effects of the ZnONPs chronic administration on the contractile activity in aortic rings (Figure 4). These assays are widely used to understand the mechanisms that regulate systemic blood pressure. Vasorelaxation contributes to a decrease in the blood pressure and vasoconstriction contributes to an increase in blood pressure. The balance of both processes determines systemic blood pressure. It is well documented that the endocannabinoid system plays an important role in the regulation of the blood pressure and cardiovascular diseases. Activation of the CB_1_ receptor, with agonists such as anandamide, induces vasorelaxation [33,34] and a decrease in the blood pressure in normotensive and hypertension models [35,36]. Blockade of the CB_1_ receptor decreases the atherosclerosis risk while activation of the CB_2_ receptor decreases the progression of the atherosclerosis acting as a cardioprotective agent [37,38,39,40,41]. Furthermore, increased CB_1_ receptor expression has been associated with vascular smooth muscle cell proliferation and atherosclerosis [42,43]. ACPA, a selective CB_1_ receptor agonist, has been reported to induce aortic vasorelaxation in previous studies [44]. In the present study, the treatment with ZnONPs modified the ACPA-induced vasorelaxation response even eliciting vasoconstriction (Figure 4a). Phenylephrine-induced vascular contraction is known to be mediated by mechanisms that allow calcium influx into smooth muscle cells through of calcium channels and other transporters [45]. ACPA has previously been reported to induce vasorelaxation through a mechanism mediated by activation of the calcium activated potassium channel subunit alpha-1 (K_Ca_1.1) and blocking of the calcium channel, voltage-dependent, L type, alpha 1C subunit (Ca_v_1.2) [44]. It has also been reported in other cells that ZnONPs increase the concentration of cytosolic calcium through a mechanism mediated by L-type calcium channel activation [46], and this effect could be inhibiting the vasorelaxation induced by ACPA seen in control conditions. Thus, ZnONPs could be eliciting vasoconstriction through of L-type calcium channels activation, allowing calcium influx into the cell. Additionally, it has also been reported that ZnONPs can induce endothelial dysfunction [47], which means it is possible that the ZnONPs are also impairing the mechanism of synthesis and release of nitric oxide (NO) at endothelial level which play a key role in the vasorelaxation [48]. The above mechanisms could be a possible explanation for the fact that ACPA did not increase vasorelaxation despite the increase in the CB_1_ receptors expression induced by ZnONPs (Figure 5a and Figure 6). In the case of HU308, a selective CB_2_ receptor agonist elicited vasoconstriction in healthy rats, while in ZnONPs-treated rats the vasoconstrictor response was completely abolished (Figure 4b). Little is known about the mechanism through which H308 modulates vascular tone through the CB_2_ receptor. A study carried out in a cell line with high expression of the CB_2_ receptor showed that HU308 increases cytosolic calcium by two mechanisms: one acting at the level of reservoirs such as sarcoplasmic reticulum increasing the release of calcium, and another by activation of calcium channels in the cell membrane mediated by G coupled receptors (Gαq/11), and phospholipase C (PLC) way [49]. These mechanisms that increase the availability of cytosolic calcium could be responsible for the vasoconstriction effect of HU308 observed in our experiments, and if this mechanism were responsible, then the decrease in the expression of CB_2_ receptors shown in this study (Figure 5b) could be associated with the reduction of vasoconstriction observed in the aorta of rats treated with ZnONPs. However, future studies are necessary to elucidate these mechanisms in this artery.

ZnONPs increased CB_1_ receptors expression and decreased CB_2_ receptor expression in the aorta wall (Figure 5). At present, it is debated whether the expression of these receptors is correlated with the progress of atherosclerosis, specifically the CB_1_ receptor as atherogenic and the CB_2_ receptor as atheroprotective [15,16,17,18,42,43]. Our study shows a correlation of increased expression of the CB_1_ receptor and decreased CB_2_ receptor with the development of atherosclerosis. However, future studies are needed to elucidate the molecular signaling mechanism by which increased expression of the CB_1_ receptor induces atherosclerosis.

Finally, the current study shows that ZnONPs induced an increase in the systolic blood pressure in healthy rats. To our knowledge, it is the first report of this effect. Some studies have analyzed the effect of ZnONPs on cardiovascular function but not on blood pressure [50,51,52,53]. In a clinical trial, healthy adults were exposed to inhalation of ZnONPs for a short period and no effects were found on heart rate and electrocardiographic patterns [50]. In contrast, in isolated cardiomyocytes, ZnONPs induce some electrophysiological alterations such as a decrease in beat rate and spike amplitude [51]. Moreover, it is known that atherosclerosis is associated with increased blood pressure [52,53].

## 5. Conclusions

Chronic administration of ZnO nanoparticles by oral route induced dyslipidemia and atherosclerotic lesions, which was accompanied by alterations at the structural level, CB_1_ and CB_2_ receptor expression, vascular contractility, and increased blood pressure. ZnONPs-induced atherosclerosis is associated with increased expression of the CB_1_ receptor and decreased expression of the CB_2_ receptor in the aorta wall.

## Figures and Tables

**Table 1:** Lipid profile and atherosclerosis risk.

Experimental Group	Total Cholesterol mg/dL	HDL-C mg/dL	LDL-C mg/dL	Triglycerides mg/dL	Atherogenic Index
Control	52 ± 2	23 ± 2	38 ± 3	32 ± 2	0.14 ± 0.04
ZnONPs- 1 month	46 ± 3	21 ± 1	19 ± 2 ^a^	48 ± 3 ^a^	0.34 ± 0.04 ^a^
ZnONPs- 2 moths	57 ± 3	31 ± 4	36 ± 2	43 ± 3 ^a^	0.15 ± 0.07
ZnONPs- 3 months	67 ± 6 ^a^	40 ± 2 ^a^	29 ± 3 ^a^	31 ± 1	0.00 ± 0.11

^a^ statistically significant regarding the control (*p* < 0.05).

**Table 2:** Effects of oral administration of ZnONPs on systolic blood pressure in healthy rats.

Systolic Blood Pressure (mmHg)			
Control	Rats treated with ZnONPs		
	One-month	Two-months	Three-months
116 ± 3	125 ± 9	129 ± 4	137 ± 4 ^a^

^a^ There is a significant difference compared to the control group with *p* < 0.05.

## Data Availability

Data are contained within the article.
